# Exploring the Use of the Behavior Change Technique Taxonomy and the Persuasive System Design Model in Defining Parent-Focused eHealth Interventions: Scoping Review

**DOI:** 10.2196/42083

**Published:** 2023-06-21

**Authors:** Mindy Silva, E Jean Hay-Smith, Fiona Graham

**Affiliations:** 1 Rehabilitation Teaching and Research Unit University of Otago Wellington New Zealand

**Keywords:** persuasive technology, behavior change, parent-focused eHealth, Capability, Opportunity, and Motivation–Behavior, COM-B, Fogg Behavior Model, FBM, behavior intervention, publications, effectiveness, usability, active ingredient, scoping review, mobile phone

## Abstract

**Background:**

Taxonomies and models are useful tools for defining eHealth content and intervention features, enabling comparison and analysis of research across studies and disciplines. The Behavior Change Technique Taxonomy version 1 (BCTTv1) was developed to decrease ambiguity in defining specific characteristics inherent in health interventions, but it was developed outside the context of digital technology. In contrast, the Persuasive System Design Model (PSDM) was developed to define and evaluate the persuasive content in software solutions but did not have a specific focus on health. Both the BCTTv1 and PSDM have been used to define eHealth interventions in the literature, with some researchers combining or reducing the taxonomies to simplify their application. It is unclear how well the taxonomies accurately define eHealth and whether they should be used alone or in combination.

**Objective:**

This scoping review explored how the BCTTv1 and PSDM capture the content and intervention features of parent-focused eHealth as part of a program of studies investigating the use of technology to support parents with therapy home programs for children with special health care needs. It explored the active ingredients and persuasive technology features commonly found in parent-focused eHealth interventions for children with special health care needs and how the descriptions overlap and interact with respect to the BCTTv1 and PSDM taxonomies.

**Methods:**

A scoping review was used to clarify concepts in the literature related to these taxonomies. Keywords related to parent-focused eHealth were defined and used to systematically search several electronic databases for parent-focused eHealth publications. Publications referencing the same intervention were combined to provide comprehensive intervention details. The data set was coded using codebooks developed from the taxonomies in NVivo (version 12; QSR International) and qualitatively analyzed using matrix queries.

**Results:**

The systematic search found 23 parent-focused eHealth interventions described in 42 articles from various countries; delivered to parents with children aged 1 to 18 years; and covering medical, behavioral, and developmental issues. The predominant active ingredients and intervention features in parent-focused eHealth were concerned with teaching parents behavioral skills, encouraging them to practice and monitor the new skills, and tracking the outcomes of performing the new skills. No category had a complete set of active ingredients or intervention features coded. The two taxonomies conceptually captured different constructs even when their labels appeared to overlap in meaning. In addition, coding by category missed important active ingredients and intervention features.

**Conclusions:**

The taxonomies were found to code different constructs related to behavior change and persuasive technology, discouraging the merging or reduction of the taxonomies. This scoping review highlighted the benefit of using both taxonomies in their entirety to capture active ingredients and intervention features important for comparing and analyzing eHealth across different studies and disciplines.

**International Registered Report Identifier (IRRID):**

RR2-doi.org/10.15619/nzjp/47.1.05

## Introduction

### Background

Taxonomies and models are useful tools for defining eHealth intervention content and delivery, enabling the comparison and analysis of research across studies and disciplines [1]. The Behavior Change Technique Taxonomy version 1 (BCTTv1) was developed to decrease ambiguity in defining specific characteristics inherent in health interventions [2], but it was developed outside the context of digital technology. In contrast, the Persuasive System Design Model (PSDM) was developed to define and evaluate the persuasive content in software solutions but did not have a specific focus on health [3]. Both the BCTTv1 and PSDM have been used to define eHealth interventions in the literature [4-9], with some researchers combining the taxonomies to include both the health and persuasive technology components [10-12]. However, there have been concerns regarding the ability of the taxonomies to capture the variability and dynamic nature of health interventions [13-15]. In addition, it is unclear how well the BCTTv1 and PSDM accurately define eHealth and whether they should be used alone or in combination. Therefore, a scoping review methodology was used to develop a relevant data set to explore how the BCTTv1 and PSDM capture the content and delivery of eHealth interventions. The scope was concerned with parent-focused eHealth as part of a program of studies investigating the use of technology to support parents with therapy home programs for children with special health care needs.

### The Taxonomies

The BCTTv1 taxonomy defines 93 distinct behavior change techniques (BCTs) categorized into 16 behavioral determinants. The behavioral determinants include categories common to many behavior change theories, such as goals and planning, social support, and shaping knowledge [2,16]. The BCT represents the smallest component of a behavior change theory that could bring about a change in behavior under the right circumstances. As such, BCTs are described as the “active ingredients” of an intervention [17].

Persuasive technology [18] refers to the use of technology, such as computers, mobile devices, and the internet, to influence and change people’s attitudes or behaviors. Although technology is often described as a service delivery vehicle in eHealth, digital applications that are responsive to a user’s input can mimic social drivers of behavior and are more persuasive than static communication media such as print [19,20]. Persuasive technology ranges from simple nudges and reminders to more complex interactive systems that use personalized feedback and rewards to motivate and engage users. On the basis of the earlier work by Fogg [18] describing persuasive technology, Oinas-Kukkonen and Harjumaa [3] proposed the PSDM grouping of 28 persuasive system design (PSD) elements into 4 design principles. A fifth design principle was later added to capture the coaching elements found in many eHealth interventions [6]. Despite being called a model, the PSDM does not define the relationships between the design elements and principles [21] and, thus, can be considered a taxonomy akin to the BCTTv1 [12].

The BCTTv1 and PSDM have a different focus; however, there is considerable overlap in the categories and principles of these 2 taxonomies. Therefore, some systematic reviewers have merged labels within the 2 taxonomies ad hoc [9-12]. However, labels within the 2 taxonomies may describe different characteristics of the same eHealth intervention. For example, *Self-Monitoring* from the BCTTv1 is a BCT that includes activities such as using a diary or checklist to self-monitor behavior. This requires some discipline and commitment to keep track of what is being monitored. In persuasive technology, *Self-Monitoring* defines an automated feature of technology that requires little effort from the user but potentially requires the user to give up a level of privacy for the convenience of having the technology do the monitoring for them. *Self-Monitoring* as captured by these 2 taxonomies clearly represents different behaviors and experiences for the user and, therefore, potentially different outcomes.

Combining multiple taxonomies to define an intervention presents a challenge in terms of efficiency and usability. Although merging taxonomies based on similar labels or constructs may improve efficiency, it could also result in the omission of important ingredients or features. Therefore, this review explores the similarities and differences between the BCTTv1 and PSDM to gain a deeper understanding of their usability individually and in combination to define the content and delivery of digital interventions in parent-focused eHealth.

## Methods

### Overview

Scoping reviews are an alternative to systematic reviews when there is a need to clarify characteristics related to a concept in the literature [22,23]. As scoping reviews are not intended to inform practice or answer effectiveness questions, they do not include a critical appraisal of the literature and cannot be used to determine intervention effectiveness [24,25]. Scoping reviews require a systematic review approach and provide a methodologically rigorous structure; however, they are intended to be iterative. The search terms and questions are refined as the researchers become immersed in the available literature [24,25]. The PRISMA-ScR (Preferred Reporting Items for Systematic Reviews and Meta-Analyses extension for Scoping Reviews) guidelines [26] accommodate changes in scoping review protocols over time and guided this scoping review report [27], along with the original framework provided by Arksey and O’Malley [24].

### Population

Parents of children with special health care needs were the population of interest in this scoping review. Children with special health care needs are defined as children with “chronic physical, developmental, behavioural, or emotional conditions who also require health and related services of a type or amount beyond that required of children generally” [28].

### Context

Many parent-focused eHealth interventions use computer-mediated interactions as a primary feature, involving mostly synchronous videoconferencing between the parent and the therapist [29]. However, persuasive technology was a key interest, and therefore, the eHealth interventions needed to predominantly include human-computer interaction to be included in this scoping review. In other words, the parents needed to interact directly with a digital application that was responsive to their input. In addition, the continuous evolution of technology highlights the importance of focusing on underlying principles that define the core functionality of eHealth interventions to facilitate research and future development [30-32]. Therefore, the context of this scoping review included all parent-focused eHealth interventions regardless of the underlying theories, application, platform, or device being used.

### Content

The search was pragmatically limited to articles published after 2008, when both the BCTTv1 and PSDM were first published [2,3], and the subsequent 10-year period in accordance with recommendations for limiting the number of articles in a scoping review [33]. This provided a comprehensive yet manageable database to examine the relationship, similarities, and differences between the BCTTv1 and PSDM, providing insights into how the active principles captured by these taxonomies could be applied in future studies.

### Objectives

The objective of this scoping review was to develop novel insights into how the BCTTv1 and PSDM, 2 increasingly popular taxonomies, identify active ingredients and features of eHealth interventions to inform future research and design.

This review answered two questions: (1) What are the active ingredients and persuasive technology features commonly found in parent-focused eHealth interventions for children with special health care needs? and (2) How do the active ingredients and features described in parent-focused eHealth literature overlap and interact with respect to the BCTTv1 and PSDM taxonomies?

### Variation From the Published Protocol

A protocol for this scoping review was published elsewhere [34]. Consistent with the iterative and responsive nature of scoping reviews [33], three variations from the protocol are noted. First, the term “ingredients and features” replaced the term “principles” to decrease ambiguity in terminology. Second, the term *persuasive eCoaching* did not replace PSDM as originally suggested. Further immersion in the data and related literature found that the elements suggested by Lentferink et al [6] did not reflect an eCoaching process.

**Figure 1:**
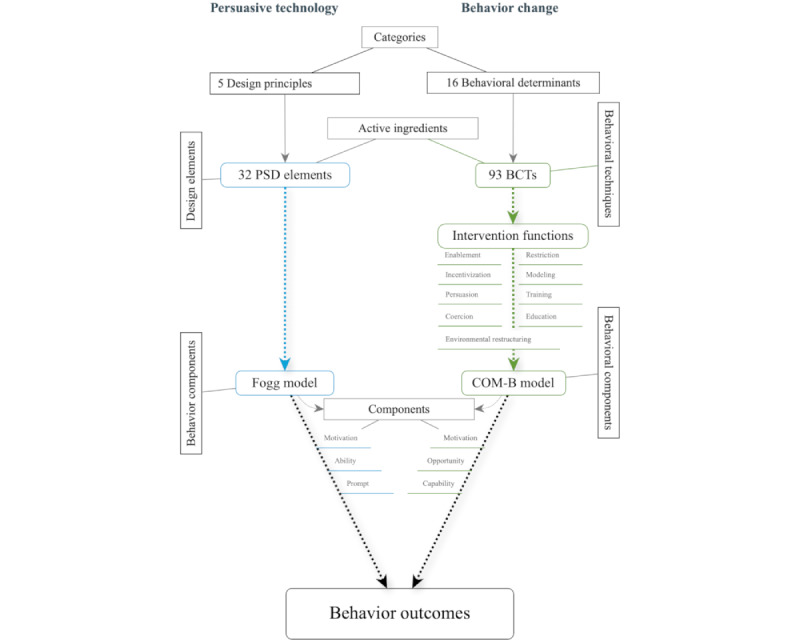
Synthesis of active ingredients, intervention features, and behavior components. BCT: behavior change technique; PSD: persuasive system design; COM-B: Capability, Opportunity, and Motivation–Behavior.

The third variation related to the illustration within the protocol depicting the relationship between the PSDM and BCTTv1. This figure has been updated (Figure 1) in light of scoping review findings. The labels within the figure were changed to more accurately represent the components of the behavior change models related to the 2 taxonomies (previously labeled *Behavior Targets*) and behavior outcomes (previously labeled *Behavior*). The additional PSD category and elements suggested to represent coaching elements of eHealth [6] were also included as PSD principles (increased from 4 to 5) and elements (increased from 28 to 32). The updated figure illustrates how the BCTTv1 and PSDM relate and link to behavioral outcomes through their related models: the Capability, Opportunity, and Motivation–Behavior (COM-B) model and the Fogg Behavior Model (FBM), respectively. Each model is made up of behavior components linked to BCTs and PSD elements that represent the behavioral determinants and design principle categories in the taxonomies. The 9 intervention functions linking BCTs to behavioral components in the BCTTv1 have also been included.

### Search Strategy

The search strategy was informed by guidelines for scoping reviews [24,26,35] and included a systematic search of MEDLINE (Ovid), Embase (Ovid), PsycINFO (Ovid), Scopus, Web of Science, CINAHL (EBSCOhost), and ERIC (Ovid) to identify potentially relevant articles. Keywords related to parent-focused eHealth were used, where the parent was the intended target of the intervention and their child’s health or behavior was the intended outcome. To be included, interventions required human-computer interaction. For the purpose of this review, human-computer interaction was conceptualized as user-generated content and an element of automation to support the parents’ behavior change. Interventions that were predominantly a replacement for face-to-face interventions or coaching (ie, computer-mediated synchronous communication), were not interactive (eg, static text, such as a digital version of an information pamphlet), or were solely intended as an education platform were excluded for this reason. In addition, as the aim of the larger study was to support parents, interventions that predominantly required the child rather than the parent to interact with the technology were also excluded. A detailed list of the inclusion and exclusion criteria can be found in Table 1.

Keywords were developed and refined through an exploratory search of the literature using Scopus. Keywords, Medical Subject Heading terms, and Excerpta Medica Tree terms were tested in Scopus and then extrapolated to match the criteria of the remaining databases in consultation with a medical librarian. Keywords (detailed in Multimedia Appendix 1) were used to conduct an electronic search of MEDLINE (Ovid), Embase (Ovid), PsycINFO (Ovid), Scopus, Web of Science, CINAHL (EBSCOhost), and ERIC (Ovid). The search yielded 9405 articles published between 2009 and 2018.

**Table 1:** Inclusion and exclusion criteria.

Category	Inclusion criteria	Exclusion criteria	Rationale
Human computer interaction	The intervention included human-computer interaction delivered using the internet or mobile technology (ie, automated responses to active human engagement with the technology).	The intervention was only 1-way communication without any interaction from the user (eg, only involved SMS text messaging and reminders), was a replacement for face-to-face interventions or coaching (ie, only used synchronous communication), was not interactive (eg, only static text, such as a digital version of an information pamphlet), or only used computer-mediated communication (eg, social media without any automated elements).	PSD^a^ is specifically concerned with the relationship that technology is able to form with the user, and we were primarily interested in automated aspects of the technology that allowed the intervention to be scaled (ie, reach a large number of users) without increasing therapist contact time.
Chronic childhood disability	The intervention was aimed at parents to address health-related issues that were likely to last >6 months in their children. Examples of health-related issues might be obesity, disordered sleep, diabetes, or disability-related conditions such as cerebral palsy and autism.	The intervention was intended for a single event of medical care or to address a health-related issue of <6 months (eg, preparation for surgery, vaccinations, or short-term health conditions such as postoperative management following tonsillectomy in typically developing children).	Behavior change in the short term (<6 months) requires a different approach and has different challenges and underlying mechanisms from the long-term behavior change that is needed for chronic conditions.
Intervention Aim	The intervention was aimed at the child’s parents to support behavior change in their child at home, at school, or in their community. Teachers, other caregivers, and children may also be included in the intervention, but parents must be the primary target or at least equally targeted.	The intervention was targeted at the child. Parents’ participation was only as an adjunct to the intervention (eg, virtual reality game where the child played the game and the parent helped set it up and kept a diary of how it was used). Interventions targeting parents with health issues (eg, parental cancer and parental mental health) were excluded.	Parents play a key role in their child’s health and behavior. Our review was specifically concerned with interventions supporting parents to manage their child’s health needs. Parents with their own health needs require additional supports and approaches that were not the focus of this review.
Intervention Outcome	Outcomes of interest included the child’s health-related or behavior issues (eg, child’s mental health, behavior, fitness, diet, sleep, and biomarkers) or parent behaviors that directly addressed the child’s health issues (eg, parents giving children healthier meal options, regular bedtimes, physical activity opportunities, and less screen time).	The outcome was primarily concerned with the parents’ health-related issues or well-being or behaviors that indirectly improved children’s health (eg, decreased parental stress or parents engaging better with health services, such as attending medical appointments or a parent education class).	Our outcome of interest was the child’s health and behavior. Interventions targeting parent outcomes would have different approaches and emphases that would not meet our objectives for this review.
Age range of the children	The intervention group included parents of children aged between 2 and 12 years. Children or preadolescents are also more likely to require active support from their parents than adolescents, who can navigate transportation and leisure-time activities by themselves or with their peers, supporting the present delimitation of child age to 5-12 years [36].	The intervention was only aimed at teenagers or at infants or babies aged <2 years.	Children require different parenting approaches at different ages and stages. We limited our age group to children who were able to actively participate in an intervention but not yet at a stage of autonomy that is seen in teenagers.
Intervention Duration	The intervention was intended to be used over >1 week.	The intervention was only intended to be accessed once or twice (eg, reading information or watching a video to prepare children for a one-off surgical event).	The intervention needed to address long-term behavior change.
Study Design	All studies where the eHealth intervention was described included qualitative and quantitative research.	The eHealth intervention was not adequately described.	There needed to be sufficient details of the intervention to code the BCT^b^ and PSD elements.

^a^PSD: persuasive system design.

^b^BCT: behavior change technique.

### Screening for Eligibility

After duplicate removal, 4843 abstracts were screened stepwise at the title (n=3228, 66.65% remaining), abstract (n=136, 2.81% remaining), and full text (n=102, 2.11% remaining) levels. After duplicates were removed, titles and abstracts were imported into EndNote X7 (Clarivate Analytics). Publication titles were used to remove studies that were clearly not related to the scoping review purpose, were a summary of academic proceedings, or did not contain details of specific eHealth interventions.

The reasons for exclusion were recorded (Figure 1). As the unit of data analysis was the eHealth intervention, articles that did not have basic information describing the intervention were excluded unless there were other publications about the same eHealth intervention with sufficient details. This included pamphlets and other gray literature found through reference mining of the full-text articles. An additional 8 articles were included, bringing the final number of articles to 42.

Abstracts were screened independently by 2 study authors. All the retained articles were retrieved as full-text articles and reviewed independently by 2 authors. Throughout the process, disagreements were resolved through discussion between the 2 reviewers and, at times, a third author.

**Figure 2:**
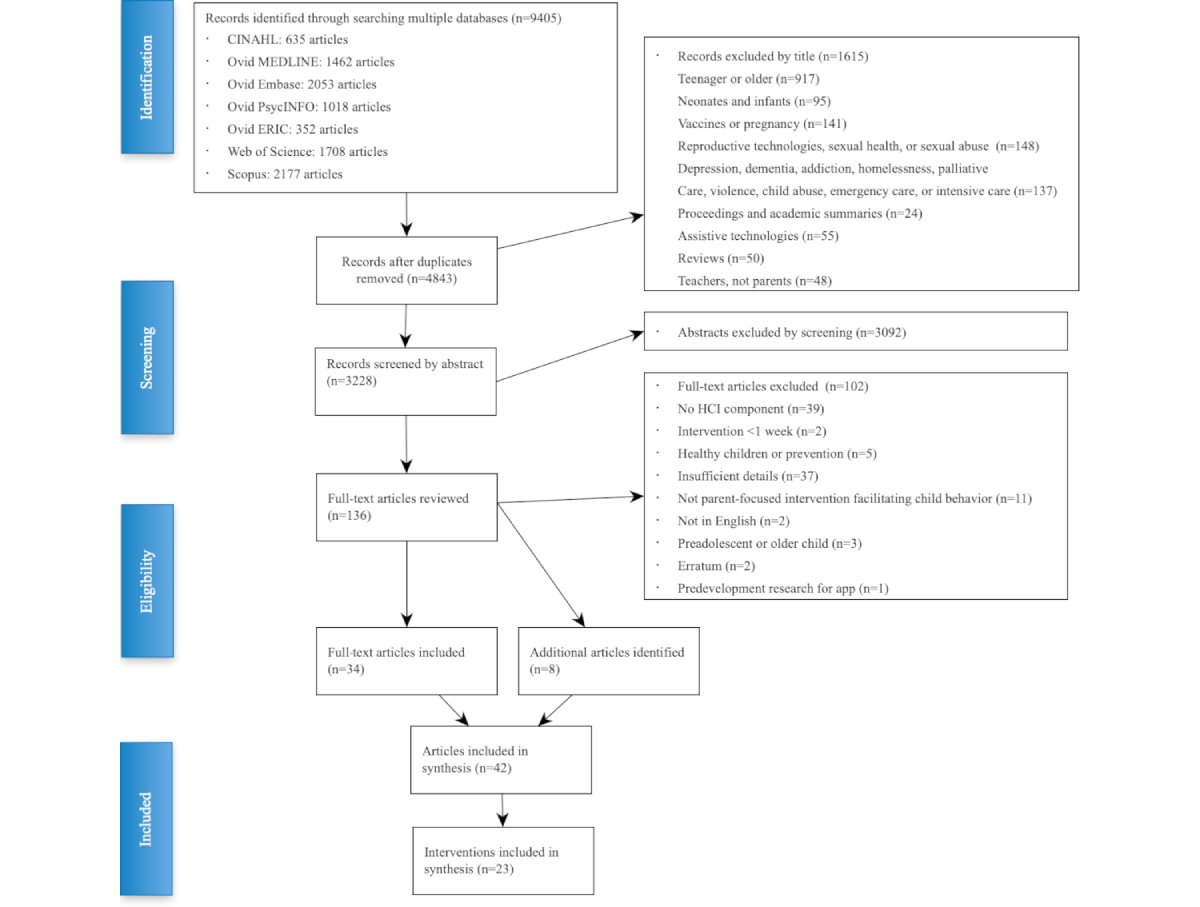
Scoping review selection process. HCI: human-computer interaction.

Publications describing the same intervention in a different country or population were collapsed into a single unit of analysis, as were publications reporting on different aspects of the same study. This resulted in 23 digital health interventions to analyze. Figure 2 illustrates the selection process using the PRISMA-ScR flow diagram [26].

### Data Extraction and Analysis

Data from multiple publications on the same study or intervention were combined, as well as information about the intervention available on the web, to extract the most comprehensive details of the eHealth intervention. Data were charted in table form for content related to year and country of publication, health conditions covered, theories mentioned, and age range of the children targeted by the interventions. The type of technology hardware used (eg, tablets and smartphones) was also recorded. Any references to the BCTTv1, the PSDM, or their related models and theories were noted.

A qualitative process of deductive content analysis [37] of the extracted data occurred in iterative cycles of coding, refining codes and recoding to define the content, patterns, and relationships of each intervention’s active ingredients and features. Articles were coded in NVivo (version 12; QSR International) with matrix-coding queries used to look for relationships and patterns. The BCTTv1 and PSDM taxonomies were used as the codebook for this analysis. Therefore, tests of codebook reliability and validity were not considered necessary as codes were not added or removed. Definitions were clarified as needed throughout the coding process. Coders had varied previous experience and training. EJHS had previous experience applying the BCTTv1 in several projects [38-40]. MS had no previous coding experience but undertook web-based training in BCT coding [41] before starting this process. Where publications provided their own list of BCTs, these supplemented the retrospective codes identified but did not replace them.

### Coding Development and Process

Initially, 4 articles were read and coded independently by MS and JHS. The codes were discussed to reach an agreement on the definition and parameters of each code. As the definitions in the PSDM are not robust, the persuasive technology text [18] and course notes from the behavior design guide workshop [42] were used to supplement the understanding of PSDM coding use. Credibility was supported via both coders coding all articles independently and then discussing codes at weekly meetings where further refinements of code definitions were made and decision rules were documented. External advice from academic colleagues with expertise in the BCTTv1 and PSDM was sought once for each taxonomy when an agreement could not be reached between the 2 primary coders. A log of decisions that contributed to the final coding scheme was kept as an audit trail. Once all articles were coded and no further updates were made to the coding manual, all articles were uploaded into NVivo, reread, and recoded based on the final coding parameters.

Coding rules were expanded beyond the BCTTv1 guidelines when there was a lack of clarity regarding which of the 2 related codes to use. For example, if it was difficult to determine whether self-monitoring was directed at the behavior or the outcome, it was coded as both *Self-monitoring of behavior* and *Self-monitoring of outcome(s)*. Knowledge and education were a large part of most interventions but were often described with insufficient detail for BCT coding requirements. Text related to education, instruction, or the provision of knowledge was not coded unless it clearly adhered to a BCTTv1 definition given that research suggests that education and knowledge on their own do not change behavior [29-31]. This contrasts with the approach taken by other authors, who coded any reference to education as *Information About Health Consequences* and *Instruction on How to Perform the Behavior* [28]. All text was coded in this way before checking the supplementary material where BCTs were detailed by the publication authors. Where supplementary material was provided listing the BCTs, this was reviewed after coding, and any additional codes were added.

NVivo queries were used to generate 3 matrix tables representing how the BCTs and PSD elements were represented in the data. The first table showed the number of times a BCT was coded with a PSD element (BCT-PSD pairs). The other 2 tables showed the number of times a BCT was coded with a different BCT (BCT-BCT pairs) or a PSD element was coded with a different PSD element (PSD-PSD pairs) in the same intervention. These matrix tables were exported to Microsoft Excel (Microsoft Corp) and enabled us to explore the relationship between BCTs and PSD elements as they were represented in the data.

**Figure 3:**
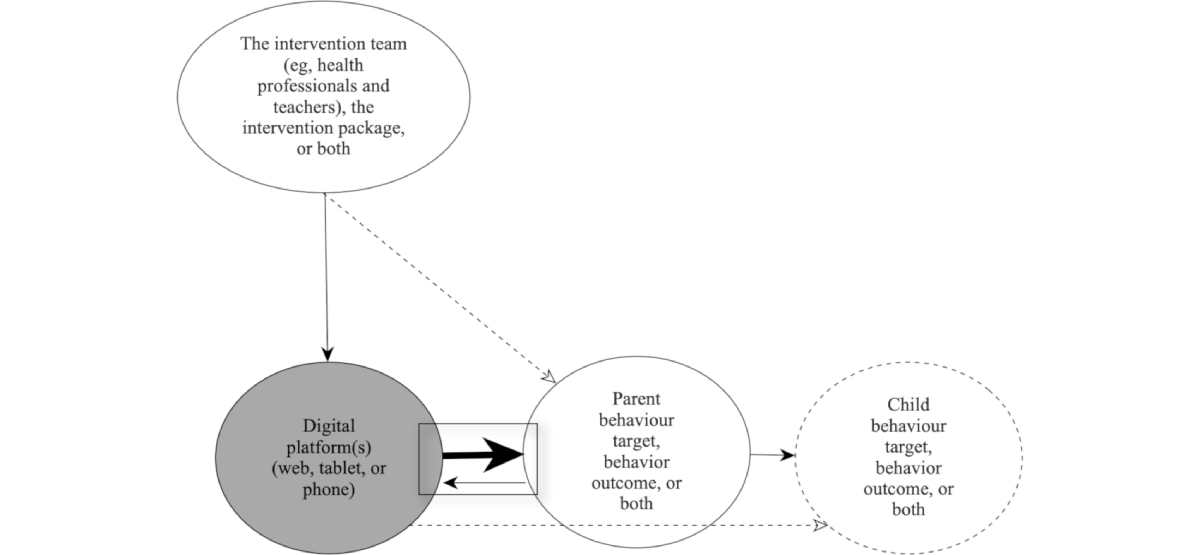
Relationships in parent-focused eHealth. The largest arrow represents the focus of this scoping review. Solid arrows represent more commonly described relationships; dashed arrows represent other relationships described.

By definition, persuasive technology requires human-computer interaction where the technology can form a “relationship” with the user. In this scoping review, technology was considered an active participant in the intervention, taking part in a “relationship” with the parent. In Figure 3, the 2 arrows in the rectangle represent this relationship in a parent-focused eHealth intervention, which was the focus of this review.

## Results

### Overview

This scoping review mapped 23 parent-focused eHealth interventions included in 42 articles published between 2009 and 2018. The most common study type in the included articles was randomized controlled trials (15/42, 36%). The next most common study types were cohort (5/42, 12%) and qualitative (4/42, 10%) studies. The publications also included mixed methods studies (2/42, 5%), co-design (2/42, 5%), case series studies (2/42, 5%), case-control studies (2/42, 5%), action research (1/42, 2%), and a randomized controlled trial protocol (1/42, 2%). The remainder of the published materials (8/42, 19%) included reports, pamphlets, and supplementary material such as tables and images of the digital intervention that were included if they detailed the intervention content for coding purposes (Table 2).

The authors of the articles were from the United States (13/42, 31%); Australia (10/42, 24%); Europe (4/42, 10%); the United Kingdom (3/42, 7%); China, New Zealand, and Sweden (2/42, 5% per country); and Belgium, Canada, Israel, Italy, Japan, and Poland (1/42, 2% per country). The parent-focused eHealth interventions were delivered to parents with children ranging in age from 1 to 18 (mean 7, SD 5) years (Table 3).

The range of chronic childhood health and behavioral concerns is listed in Table 4 and covers medical, behavioral, and developmental issues. Children with cerebral palsy and other childhood physical disabilities were not represented.

**Table 2:** Interventions included in the scoping review (n=23).

Intervention	Country	Latest publication year	Source	Study type
ALLapp (acute lymphoblastic leukemia) [43,44]	China	2016	Peer-reviewed publication	Mixed; action research
AutismPro [45]	Poland or Japan	2012	Peer-reviewed publication	Cohort
Boston Breathes [46]	United States	2015	Peer-reviewed publication	RCT^a^
BraveOnline [47-49]	Australia	2016	Peer-reviewed publication	RCT
CHESS^b^ [50]	United States	2012	Peer-reviewed publication	RCT
CLKonline [51-53]	Australia	2017	Peer-reviewed publication and pamphlet	RCT; other
e-Aster^c^ [54]	United States	2012	Peer-reviewed publication	Qualitative
eCPP^d^ [55]	United States	2015	Peer-reviewed publication	Co-design
Enhancing Interactions [56]	United States	2017	Peer-reviewed publication	Qualitative; RCT
Strongest Families FASD^e^ [57]	Canada	2015	Peer-reviewed publication	Protocol
TE-HNC^f^ [58]	United States	2016	Peer-reviewed publication	Case study
HEAL^g^ [59]	United States	2016	Peer-reviewed publication	Co-design
Health Heroes [60]	United Kingdom	2015	Peer-reviewed publication	Qualitative; action research
ICT^h^ [61]	United States	2017	Peer-reviewed publication	Cohort
ImPACT^i^ Online [62,63]	United States	2017	Peer-reviewed publication	Mixed methods
Internet-LP^j^ [64]	Australia and Belgium	2016	Peer-reviewed publication	Case-control
My Child’s Asthma [65,66]	United States	2012	Peer-reviewed publication	RCT
PMT^k^ [67]	Sweden	2014	Peer-reviewed publication	RCT; cohort
Sensory Treat [68]	Israel	2018	Peer-reviewed publication	Cohort
Time2bHealthy [69]	Australia	2017	Peer-reviewed publication	RCT
TPOL^l^ [70-72]	Australia and New Zealand	2017	Peer-reviewed publication	RCT; cohort
Trec-Lifestyle (Trento region as a citizen-controlled clinical record system) [73]	Italy	2017	Peer-reviewed publication	Qualitative
WHAAM^m^ [74-77]	Europe	2018	Peer-reviewed publication, report, and pamphlet	Case series; other

^a^RCT: randomized controlled trial.

^b^CHESS: comprehensive health enhancement support system.

^c^e-Aster: Electronic Asthma Symptom Tracking and Exacerbation Reduction.

^d^eCPP: Electronic Chicago Parent Program.

^e^FASD: fetal alcohol spectrum disorder.

^f^TE-HNC: Technology-Enhanced–Helping the Noncompliant Child.

^g^HEAL: Healthy Eating and Active Living.

^h^ICT: interactive computerized training.

^i^ImPACT: Improving Parents as Communication Teachers.

^j^LP: Lidcombe Program.

^k^PMT: parent management training.

^l^TPOL: Triple P Online.

^m^WHAAM: Web Health Application for ADHD Monitoring.

**Table 3:** Content, context, and population details of the included interventions (n=23).

Intervention	Diagnosis	Stakeholders (other than parents)	Age target (years)
ALLapp (acute lymphoblastic leukemia) [43,44]	Acute lymphoblastic leukemia	Medical staff	1-14
AutismPro [45]	Autism	N/A^a^	3-7
Boston Breathes [46]	Asthma	Care providers, physicians, and asthma specialist	9-17
BraveOnline [47-49]	Anxiety disorder (other than OCD^b^, panic disorder, or posttraumatic stress disorder)	N/A	3-17
CHESS^c^ [50]	Asthma	Nurse	4-12
CLKonline [51-53]	Child anxiety disorders	N/A	2-6
e-Aster^d^ [54]	Asthma	Care manager	10-18
eCPP^e^ [55]	Emotional and behavioral problems	N/A	Not specified
Enhancing Interactions [56]	ASDs^f^	N/A	2-6
Strongest Families FASD^g^ [57]	Challenging behavior in children with FASD	N/A	4-12
TE-HNC^h^ [58]	Early-onset DBDs^i^ and BPT^j^	N/A	3-8
HEAL^k^ [59]	Cancer	N/A	4-10
Health Heroes [60]	Childhood weight management	N/A	5-11
ICT^l^ [61]	ASDs	N/A	3-4
ImPACT^m^ Online [62,63]	ASDs	N/A	2-12
Internet-LP^n^ [64]	Early stuttering	Speech-language pathologist	3-4
My Child’s Asthma [65,66]	Asthma	N/A	2-10
PMT^o^ [67]	ODD^p^ and CD^q^—disruptive behaviors	N/A	3-12
Sensory Treat [68]	Sensory processing disorder	N/A	0-12
Time2bHealthy [69]	Childhood obesity	Dietician	2-5
TPOL^r^ [70-72]	Early-onset conduct problems	Family and teachers	2-9
Trec-Lifestyle (Trento region as a citizen-controlled clinical record system) [73]	Overweight children	N/A	7-12
WHAAM^s^ [74-77]	ADHD^t^	Teachers, classmates, health services, and providers of other professional services	Not specified

^a^N/A: not applicable.

^b^OCD: obsessive-compulsive disorder.

^c^CHESS: comprehensive health enhancement support system.

^d^e-Aster: Electronic Asthma Symptom Tracking and Exacerbation Reduction.

^e^eCPP: Electronic Chicago Parent Program.

^f^ASD: autism spectrum disorder.

^g^FASD: fetal alcohol spectrum disorder.

^h^TE-HNC: Technology-Enhanced–Helping the Noncompliant Child.

^i^DBD: disruptive behavior disorder.

^j^BPT: behavioral parent training.

^k^HEAL: Healthy Eating and Active Living.

^l^ICT: interactive computerized training.

^m^ImPACT: Improving Parents as Communication Teachers.

^n^LP: Lidcombe Program.

^o^PMT: parent management training.

^p^ODD: oppositional defiant disorder.

^q^CD: conduct disorder.

^r^TPOL: Triple P Online.

^s^WHAAM: Web Health Application for ADHD Monitoring.

^t^ADHD: attention-deficit/hyperactivity disorder.

**Table 4:** Health conditions (n=23).

Health category and diagnosis			Studies, n (%)
**Medical**			
	Cancer	2 (9)	
	Asthma	4 (17)	
	Obesity	3 (13)	
	Anxiety	2 (9)	
**Behavioral**			
	Oppositional and disruptive behavioral disorders	4 (17)	
	Fetal alcohol spectrum disorder	1 (4)	
	Attention-deficit/hyperactivity disorder	1 (4)	
**Developmental**			
	Autism spectrum disorder	4 (17)	
	Speech dysfunction	1 (4)	
	Sensory processing dysfunction	1 (4)	

### Parent-Focused eHealth Content

This scoping review mapped the theories, models, and taxonomies described in the included publications. Just over half (13/23, 57%) of the interventions mentioned a behavior change theory. Social-cognitive theory was the most frequently mentioned (6/23, 26%), followed by self-determination theory (3/23, 13%). Only 9% (2/23) of the interventions referenced PSD (CLKonline and Strongest Families FASD), and only 4% (1/23) of the interventions (Health Heroes) referred to the Behavior Change Wheel [78] as the overarching theory for the COM-B model and the BCTTv1. This was also the only publication that included supplementary information listing the BCTs in the intervention. No articles referenced the FBM.

Some interventions were established treatment approaches adapted for a web-based medium, for example, BraveOnline [48] and TPOL [70]. Other interventions were novel approaches designed specifically for the eHealth intervention, such as Sensory Treat [68] and Health Heroes [60]. Most interventions (19/23, 83%) used a web-based platform, with 47% (9/19) of these also including a mobile phone or tablet. The remaining 17% (4/23) of the interventions were stand-alone mobile phone or tablet apps. The most popular features included in the interventions were interactive questionnaires and charts (17/23, 74%), followed by instructional videos (14/23, 61%).

Consistent with a focus on parents, all the included eHealth interventions (23/23, 100%) had the parent as the primary user, with the child (either the child’s behavior, health, or both) as the outcome target. The parent interacted directly with the digital platform, which could be linked to an intervention team, a preprogrammed intervention package (eg, a digital app), or a combination (Figure 3). Thus, the active ingredients and intervention features (ie, BCTs and PSD elements) coded in this review were those directed toward the parent, whose behavior change affected their child’s health and behavioral outcomes. However, multiple relationships could exist in any one intervention such as targeting the child or other family members as intervention users. In addition, intervention descriptions in the publication text were often focused on the parent-child interaction rather than describing the human-computer interaction between the digital intervention and the parent.

The following extract from the scoping review data set illustrates how this attention to who was being targeted influenced the coding process. In an eHealth intervention supporting parents in implementing activity schedules for children with autism spectrum disorder, the following text was coded:

The training module consisted of the following components: (a) introduction to activity schedules and their format, (b) beneﬁts of an activity schedule, (c) preparing and setting up the learning environment, (d) conducting a brief multiple-stimulus-without-replacement (e) instructional prompting and prompt fading of an activity schedule, and (f) data collection and data-based decision-making [61].

If the child were the target, coding would have captured techniques and elements such as *Prompts and Cues*, *Restructuring the Environment*, and *Information* *and* *Problem Solving*. However, as the directional relationship was from the digital intervention to the parent, this extract was coded as the BCT *Instructions on How to Perform a Behaviour* and the PSD element *Educational Coaching*.

### Active Ingredients and Intervention Features: BCTs and PSD Elements

#### Overview

The total PSD elements and BCTs coded using NVivo were analyzed by generating NVivo matrix queries to explore the patterns of BCT and PSD use visually. Microsoft Excel graphs were generated from the data. Commonly used codes and categories of the BCTTv1 and PSDM (density and frequency) were explored, along with how the use of theory influenced the active ingredients and intervention features. We referred back to the original definitions of persuasive design principles [18] to compare elements and techniques that have been suggested as appropriate to merge [10, 12]. We found the data coded with each BCT or PSD element to be conceptually different, and no BCTs or PSD elements were found to always represent the same construct. Table 5 provides one example from the dataset comparing a BCT and PSD element that has previously been suggested [12] as appropriate to merge.

**Table 5:** Comparison of the persuasive system design element Tunneling and the behavior change technique (BCT) Instruction on How to Perform a Behavior.

Label	Definition	Coded example from the data set
Tunneling	PSDM^a^ description: “system should guide users in the attitude change process by providing means for action that brings them closer to the target behavior” [3] Fogg description: “giving up a certain level of self-determination...for users, tunnelling makes it easier to go through a process [by removing choices]...for designers, tunnelling controls what the user experiences [providing a predetermined sequence of events and forced choices]” [18]	“Parents received automated feedback regarding whether their answers were correct or incorrect within the module. If a question was answered incorrectly, the parent was required to review the relevant content slides and answer the question correctly before proceeding to the next slide.” [ICT^b^]
BCT 4.1: instruction on how to perform a behavior	“Advise or agree on how to perform the behavior (includes ‘Skills training’)” [41]	“...instructional videos show children using asthma medication and peak-flow devices.” [CHESS^c^]

^a^PSDM: Persuasive System Design Model.

^b^ICT: interactive computerized training.

^c^CHESS: comprehensive health enhancement support system.

#### Density and Frequency of Active Ingredients and Intervention Features

**Figure 4:**
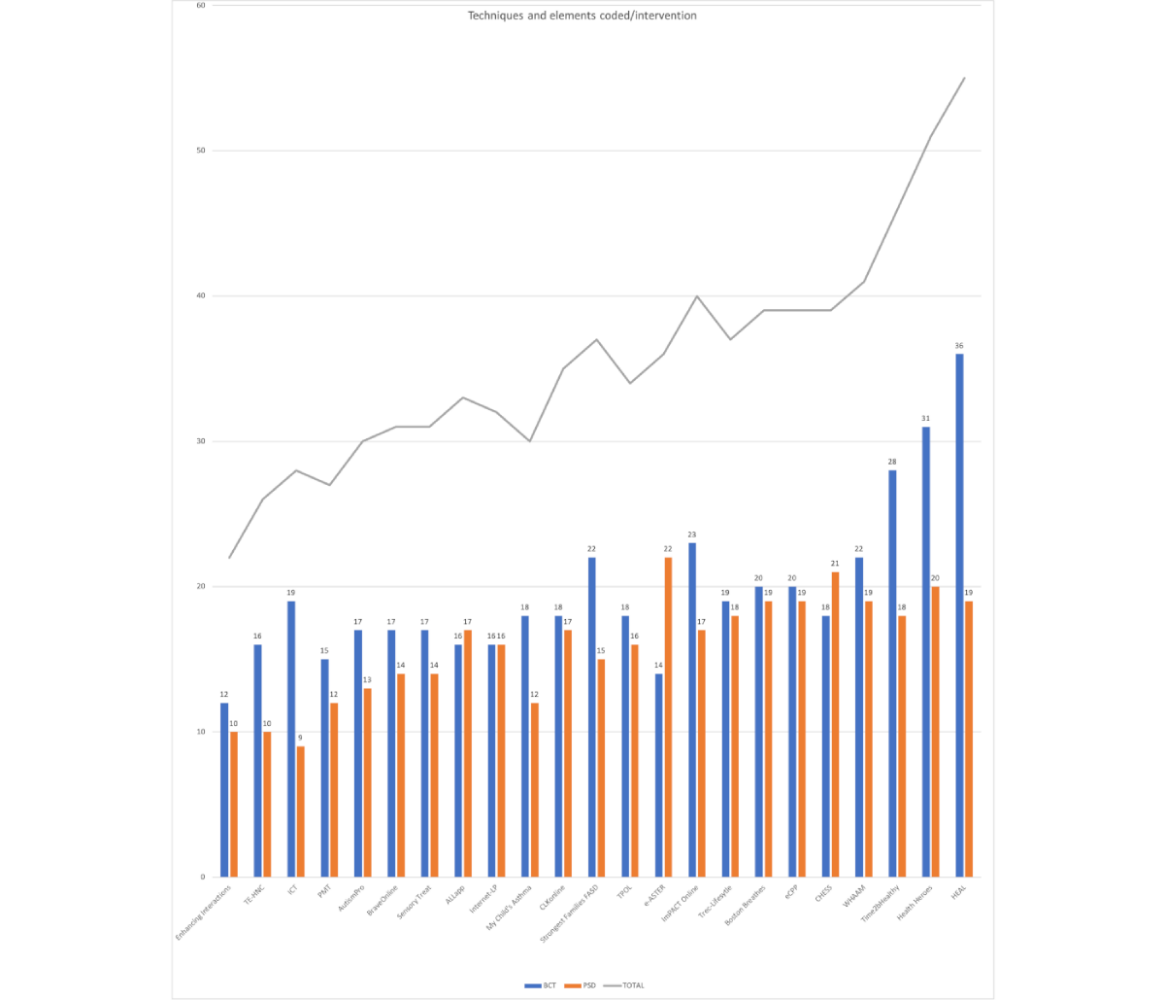
Coding density: active ingredients and intervention features coded per intervention. BCT: behavior change technique; CHESS: comprehensive health enhancement support system; e-ASTER: Electronic Asthma Symptom Tracking and Exacerbation Reduction; eCPP: Electronic Chicago Parent Program; FASD: fetal alcohol spectrum disorder; HEAL: Healthy Eating and Active Living; ICT: interactive computerized training; ImPACT: Improving Parents as Communication Teachers; LP: Lidcombe Program; PMT: parent management training; PSD: persuasive system design; TE-HNC: Technology-Enhanced–Helping the Noncompliant Child; TPOL: Triple P Online; WHAAM: Web Health Application for ADHD Monitoring.

Figure 4 shows how many active ingredients and intervention features were included in an intervention (coding density). A BCT or PSD element was counted once per intervention even if it was mentioned in several sections of the publication, in the supplementary material, or in 2 separate publications on the same intervention. Similarly, if the BCT or PSD element was implemented in different ways, it was also only counted once. For example, if the BCT *Feedback on behavior* was represented by a feedback graph and an evaluative email for the same intervention, it was counted once.

As there are 93 BCTs compared with 32 PSD elements, the interventions with the highest number of BCTs coded also had the most active ingredients and intervention features (combined BCTs and PSD elements) coded in total.

The BCTs most frequently represented were for learning a behavioral skill, practicing it, and self-monitoring the behavior and its outcomes; codes were *Behavioural Practice* and *Instructions on How to Perform a Behaviour*, *Self-Monitoring (Behaviour)* and *Self-Monitoring (Outcomes*; 17/23, 74% each), followed closely by *Demonstration of the Behaviour* and *Action Planning* (16/23, 70% each).

The PSD element most frequently coded was *Tailoring* the approach to parents was observed in every intervention (23/23, 100%). *Educational Coaching* (22/23, 96%), *Personalisation* (21/23, 91%), and *Suggestion* (20/23, 87%) were also highly represented.

The intervention My Child’s Asthma [65,66] was the only intervention that was coded for the PSD element goal setting. In this intervention, parents were supported in setting goals regarding asthma controller use and other aspects of asthma care by the system providing a list of suggested goals. Goals were populated in response to the parents’ previous reports on adherence, outcome expectations, and self-efficacy. For example, if parents reported not believing that controllers worked as the primary reason why they were not using them, they were presented with information about asthma controller efficacy. If parents believed that controllers worked but reported that they could not remember to use them, they were provided with goals related to remembering to dispense controllers more consistently.

#### Density and Frequency of Categories

Both the BCTTv1 and PSDM use broader categories (behavior determinants and design principles, respectively) to group the BCTs and PSD elements. The BCTTv1 has 16 categories, and the PSDM has 5. A matrix table was used to count the number of active ingredients and features coded for each intervention per category. Table 6 provides an example of the BCTTv1 category *Goals and Planning* from this matrix table. Each column shows the number of interventions that included an active ingredient. Each row shows the number of active ingredients and features coded per intervention.

Table 6 illustrates how the category Goals and Planning was frequently represented in the data set, with 91% (21/23) of the interventions having at least one active ingredient in this category, although very few of the active ingredients from the Goals and Planning category were actually coded in the data set. The table shows that BCTs 1.5 to 1.9 were coded in less than a quarter of the interventions, with Behavioral Contract not being coded in any. Therefore, the density of active ingredients was low, with only 29% of the possible active ingredients in this domain coded in the full data set.

**Figure 5:**
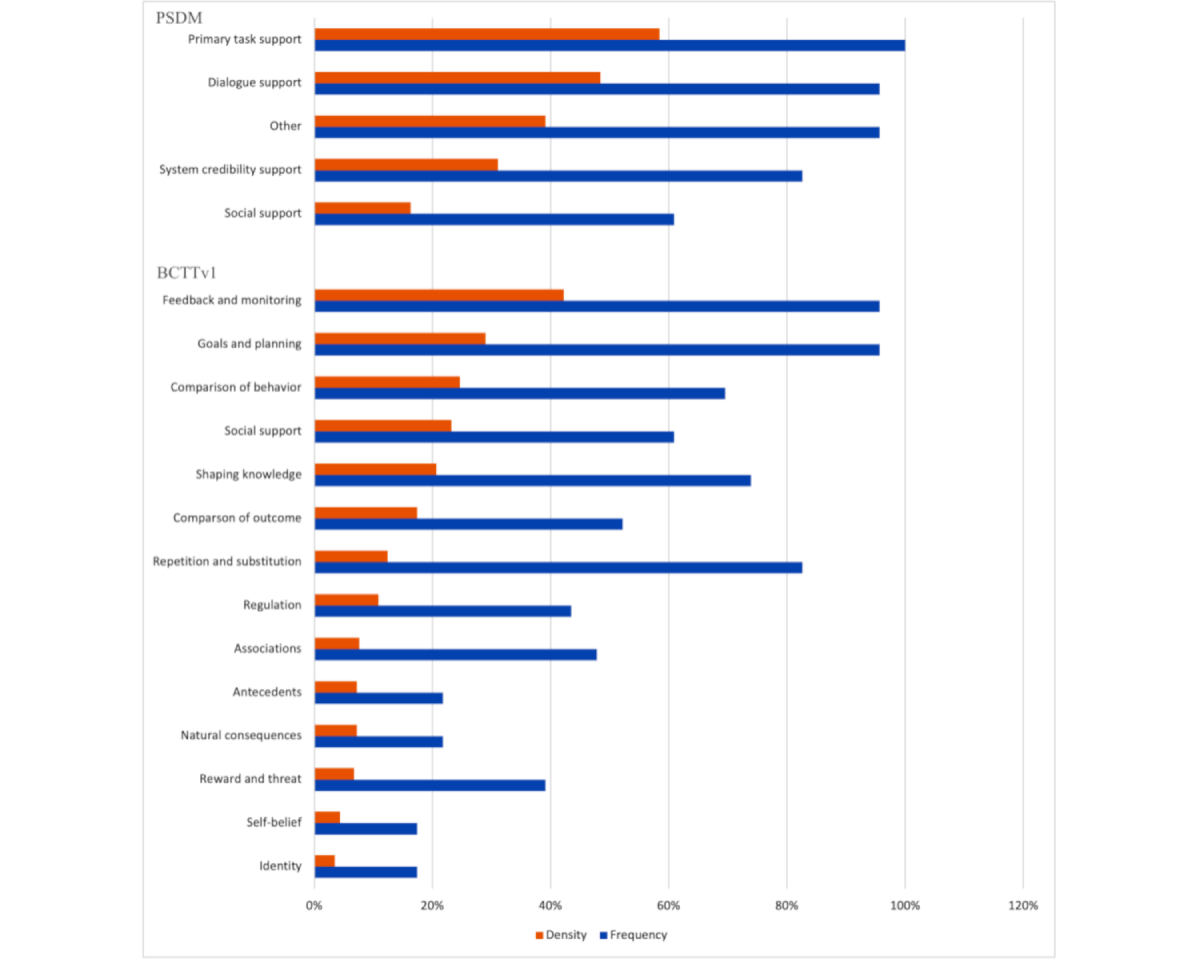
Frequency of category representation in the data set versus density of ingredient and element representation per category. BCTTv1: Behavior Change Technique Taxonomy version 1; PSDM: Persuasive System Design Model.

Figure 5 illustrates the coding frequency and density across all the BCTTv1 and PSDM domains. A total of 43 BCTs from the full set of 93 were not coded in the data set. This included all the BCTs from 2 BCTTv1 categories (*Scheduled Consequences* and *Covert Learning*) that were therefore not represented at all in the data set. All the PSDM categories were represented by at least one active ingredient coded in the data set. A total of 94% (30/32) of the PSD elements were coded at least once. The 6% (2/32) of PSD elements that were not coded (*Social Comparison* and *Recognition*) were from the *Social Support* category. No category had a complete set of active ingredients and intervention features within that category coded in the data set. *Task Support* was the most densely coded category in the PSDM (97/161, 60.2%), and *Feedback and Monitoring* was the most densely coded category in the BCTTv1 (71/161, 44.1%).

Coding by category was not representative of the active ingredients and features used in an intervention. For example, the BCTTv1 category *Repetition and Substitution* was the third most frequently represented BCTTv1 category (20/23, 87%); however, only 1 BCT (*Behavioural Practice*) was frequently coded (17/23, 74%). The only other technique coded from this category (*Graded Tasks*) was observed in 13% (3/23) of the interventions. The remaining 5 active ingredients in the *Repetition and Substitution* category were not coded at all in the data set, making it a high-frequency (19/23, 83%), low-density (20/161, 12.4%) category. Similar patterns were observed across the PSDM categories, as shown in Figure 5.

**Table 6:** Intervention code mapping table excerpt^a,b^.

Intervention	Category 1: goals and planning									
	1.1: Goal setting (behavior), n	1.2: Problem-solving, n	1.3: Goal setting (outcome), n	1.4: Action planning, n	1.5: Review behavior goal, n	1.6: Discrepancy between goals and current behavior, n	1.7: Review outcome goal, n	1.8: Behavioral contract, n	1.9: Commitment, n	Active ingredients or intervention features, n
ALLapp	0	0	0	1	0	0	0	0	0	1
AutismPro	4	3	2	2	0	0	0	0	0	4
Boston Breathes	0	0	0	1	0	0	0	0	0	1
BraveOnline	0	0	0	0	0	0	0	0	0	0
CHESS^c^	0	1	0	1	0	0	0	0	0	2
CLKonline	0	3	1	2	0	0	0	0	0	3
e-ASTER^d^	0	0	0	0	0	0	0	0	0	0
eCPP^e^	1	4	0	2	0	0	0	0	0	3
Enhancing Interactions	0	1	1	2	0	0	0	0	0	3
Strongest Families FASD^f^	0	2	0	0	0	0	0	0	0	1
TE-HNC^g^	0	1	0	0	0	0	0	0	0	1
HEAL^h^	2	4	0	2	0	0	0	0	0	3
Health Heroes	3	0	0	0	1	0	0	0	3	3
ICT^i^	0	1	0	0	0	0	0	0	0	1
ImPACT^j^ Online	1	0	1	1	0	0	0	0	0	3
Internet-LP^k^	0	2	0	1	0	0	0	0	0	2
My Child’s Asthma	4	1	0	1	2	1	1	0	0	6
PMT^l^	0	0	0	1	0	0	0	0	1	2
Sensory Treat	1	2	1	4	0	0	0	0	0	4
Time2bHealthy	7	2	1	3	1	2	1	0	0	7
TPOL^m^	3	2	1	1	0	0	0	0	0	4
Trec-Lifestyle	0	0	0	0	1	1	0	0	0	2
WHAAM^n^	1	0	3	10	0	0	1	0	0	4

^a^Density: percentage of codes used (number of behavior change techniques [BCTs] coded over 23 interventions × number of BCTs per category; 29%).

^b^Frequency: percentage of interventions that used a BCT from Goals and planning (21/23, 91%).

^c^CHESS: comprehensive health enhancement support system.

^d^e-ASTER: Electronic Asthma Symptom Tracking and Exacerbation Reduction.

^e^eCPP: Electronic Chicago Parent Program.

^f^FASD: fetal alcohol spectrum disorder.

^g^TE-HNC: Technology-Enhanced–Helping the Noncompliant Child.

^h^HEAL: Healthy Eating and Active Living.

^i^ICT: interactive computerized training.

^j^ImPACT: Improving Parents as Communication Teachers.

^k^LP: Lidcombe Program.

^l^PMT: parent management training.

^m^TPOL: Triple P Online.

^n^WHAAM: Web Health Application for ADHD Monitoring.

### Pairing of BCTs and PSD Elements

Next, we examined the overlap or interaction between BCTs and PSD elements using the BCT-PSD matrix table. An example of this table is illustrated in Table 7, which is a snapshot of the full BCT-PSD matrix table. For example, the BCTs goal setting, action planning, and problem-solving were frequently paired with PSD elements reduction, suggestions, and educational coaching. However, we observed that the more times each ingredient was coded, the greater the “scatter” of the BCT-PSD pairings. For example, the BCT *Instructions on How to Perform a Behaviour* was coded with a PSD element 142 times, representing 20 different PSD elements. Similarly, the PSD element *Tailoring* was paired with a BCT 200 times during the coding process, and these pairings included 33 different BCTs.

**Table 7:** Behavior change technique (BCT)–persuasive system design (PSD) element matrix table snapshot^a^.

			PSDM^b^ (primary task support)												
			Reduction, n		Tunnelling, n		Tailoring, n		Personalization, n		self-monitoring, n		Simulation, n		Rehearsal, n
**BCTTv1^c^ (goals and planning)**															
	1.1. Goal setting (behavior)	16		1		12		8		4		0		0	
	1.2. Problem solving	4		0		7		4		0		0		1	
	1.3. Goal setting (outcome)	6		1		3		3		0		0		0	
	1.4. Action planning	16		3		13		11		4		0		3	
	1.5. Review behavior goals	0		0		1		2		4		0		0	
	1.6. Discrepancy between current behavior and goal	1		0		1		1		1		0		0	
	1.7. Review outcome goals	1		0		0		1		1		0		0	
	1.9. Commitment	2		3		2		0		0		0		0	
**BCTTv1 (feedback and monitoring)**															
	2.1. Monitoring of behavior by others without feedback	0		1		0		2		1		0		0	
	2.2. Feedback on behavior	12		4		8		13		14		0		0	
	2.3. Self-monitoring of behavior	25		3		11		11		6		0		1	
	2.4. Self-monitoring of outcomes of behavior	17		3		11		13		6		0		0	
	2.7. Feedback on outcomes of behavior	7		1		10		15		15		0		0	

^a^BCT: PSD matrix table snapshot: n=number of times each PSD element was paired with each BCT.

^b^PSDM: Persuasive System Design Model.

^c^BCTTv1: Behavior Change Technique Taxonomy version 1.

## Discussion

### Principal Findings

#### Overview

The aim of this scoping review was to explore how the BCTTv1 and PSDM define parent-focused eHealth individually and in combination. Active ingredients and intervention features (BCTs and PSD elements) were coded in a data set of parent-focused eHealth literature and used to explore how these 2 taxonomies overlap and interact. The predominant active ingredients and intervention features found in parent-focused eHealth were concerned with teaching parents behavioral skills, encouraging them to practice and monitor the new skills, and tracking the outcomes of performing the new skills. The 2 taxonomies conceptually captured different constructs even when their labels appeared to overlap in meaning. In this section, the importance of clarifying directional relationships and intended targets before coding is highlighted first as an essential consideration. Coding accuracy and the implications of merging or condensing codes are then discussed, and the design implications for parent-focused eHealth are explored.

#### Directional Relationship, Active Ingredients, and Intervention Features in Parent-Focused eHealth

The focus of the interventions described in the parent-focused eHealth data set was primarily on the child despite the intervention’s intention to elicit a desired behavior from the parent. Parenting behaviors were often poorly defined or merely implied in the text. The importance of identifying the intended direction of interaction in a parent-focused intervention to improve reporting and coding accuracy was apparent in this scoping review analysis and has been similarly noted in another recent systematic review of digital behavior change interventions in children with chronic health conditions [79]. The target of the human-computer interaction (ie, the directional relationship) matters in parent-focused eHealth research, where each member of the triad of parent, child, and intervention participants interacts differently with the intervention. Therefore, in parent-focused eHealth, where the intervention targets the parent to improve child outcomes, careful attention to the directional relationship of the active ingredient or intervention feature being described is needed to mitigate errors in coding.

#### Active Ingredients and Intervention Features Related to Theories, Models, and Taxonomies

In this scoping review, the number of parent-focused eHealth interventions that referenced a theoretical basis or model underpinning their design (14/23, 61%) was similar to that of other published reviews. On average, approximately half of all publications in the health behavior change and persuasive technology literature have an explicit theoretical basis [6,80,81]. Similar to this scoping review, a previous systematic quantitative content analysis [80] reported that social-cognitive theories were referenced most often (7/14. 50%), followed by self-determination theories (3/14, 21%). Only 13% (3/23) of the interventions included in this scoping review mentioned either the PSDM or the BCTTv1 and the COM-B. None mentioned the FBM. Therefore, there were not enough data for any conclusions to be drawn on the existing use of these particular taxonomies in parent-focused eHealth.

Discrepancies in coding clarity and accuracy are a known issue resulting from the limited space for comprehensive reporting of the active ingredients and intervention features, ambiguity in the description of the intervention, or coders’ skill [13,14]. This was highlighted during the coding process with the Health Heroes intervention [60]. As the only intervention with a supplementary list of BCTs provided, it enabled us to compare our retrospective coding with the researchers’ list of BCTs. We found that some of the active ingredients listed in the supplementary material were not clearly described in the text, and our interpretation of the text during our retrospective coding did not always match the provided list of BCTs. This points to the importance of having supplementary lists provided by the intervention designers to improve coding accuracy in research. However, retrospective coding was still found to be useful as we identified several BCTs that were not mapped in the supplementary material. It has been noted that features may have more than one function that may not have been anticipated in the original intervention design [60], and the combination of ingredients and elements may interact in ways that may increase or decrease the effectiveness of an eHealth intervention [82,83]. Therefore, both prospective and retrospective coding may be necessary to comprehensively identify intervention ingredients for clarity and accuracy in reporting and research.

#### Merging and Simplifying Taxonomies for Coding Efficiency

Some reviews have simplified the coding of eHealth by using the categories rather than the BCTs or PSD elements. There are 16 behavioral determinants and 5 design principles as compared with the 93 BCTs and 32 PSD elements. We found that categories did not always represent the active ingredients and intervention features in an intervention well. An intervention could have many BCTs or PSD elements or only 1 element representing a category, providing an inaccurate representation of what ingredients or features actively contributed to an intervention effect. Coding by category could miss important active ingredients and intervention features and negatively affect future research related to adapting or understanding intervention effectiveness.

Combining elements and techniques in the PSDM and BCTTv1 to create a merged taxonomy has also been noted in the literature to improve coding efficiency [10,12], for example, merging the PSDs elements *Similarity, Personalisation*, and *Liking* into 1 code or merging the PSD element *Tunnelling* with the BCT *Instructions on How to Perform a Behavior.* Our coding analysis did not support this approach. No BCTs or PSD elements were found to always represent the same construct, particularly when referring back to the original taxonomy definitions [18].

Instructions on using the BCTTv1 [41] specifically caution against coding based on a name rather than a description. Similar instructions are missing for the PSDM, which could improve coding accuracy for researchers, particularly as words commonly used to describe eHealth features in the text do not necessarily represent the intended construct represented by that word in the PSDM. For example, *Personalisation* and *Tailoring* are words used interchangeably but have different definitions in the PSDM. The following text was coded as *Personalisation* despite being described as tailoring:

Portions of the practice assignments are tailored for each parent using the program in which they can choose what behaviours and strategies they will practice with their child—eCPP (electronic Chicago Parent Program) [54].

This example highlights the need for clear instructions to standardize retrospective coding using taxonomies. The original conceptual article for the PSDM [3] only provides short definitions for PSD elements. Other authors have suggested that PSD elements be coded only when there is evidence of human-computer interaction related to the description, for example, only coding *Dialogue Support* when the technology provides a praising message, not when a feedback message from a care provider contains praise [5]. As we also applied this rule to our coding, the PSD element *Goal setting* was only coded when an interactive activity with the computer assisted in setting the goal. Only 4% (1/23) of the interventions out of the whole data set included a PSD element for goal setting (Multimedia Appendix 2). In comparison, goal setting from the BCTTv1 was coded in nearly half of the interventions (*Goal setting [behavior]*: 10/23, 43%; *Goal setting [outcome]*: 8/23, 35%; Multimedia Appendix 3).

These findings suggest that taxonomies should not be merged based on similar labels or assumed constructs. Using goal setting as an example, the benefits of individualizing goals (BCT) over preselected goals (PSD) cannot be assumed. The literature points toward some parents finding it stressful to imagine goals for their child and preferring preselected goals [84].

We found constructs that appeared similar but used different labels and codes that used similar labels but represented different constructs. However, no labels within or between the taxonomies were consistently coded together. Similar-sounding labels or constructs represent different user experiences that may support different outcomes. Coding with broader categories or merging labels and constructs between BCTTv1 and PSDM taxonomies may improve efficiency but miss important differences in researching and understanding the effect of active ingredients and features in interventions.

#### Commonly Found Active Ingredients and Intervention Features in Parent-Focused eHealth

Commonly used ingredients and features do not necessarily represent the most effective active ingredients and features. In this scoping review, the most common PSD elements coded were *Tailoring, Personalisation, eCoaching, Suggestion*, and *Reduction*. *Educational Coaching* (providing knowledge about the target behavior) and *Suggestion* (suggesting that users carry out the target behavior) were also frequently included. The most frequent BCTs coded addressed advice and demonstration on how to perform a behavior, prompted practice or rehearsal of a behavior, and monitored and provided feedback on behavior and action planning. This implies that digital interventions addressing parent-focused eHealth are tailored toward family needs and interests, simplifying tasks and individualizing the intervention. The predominant active ingredients and intervention features in parent-focused eHealth were concerned with teaching parents behavioral skills, encouraging them to practice and monitor the new skills, and tracking the outcomes of performing the new skills to improve skill and capacity in parents supporting their children’s health and behavior needs. In a separate systematic review of health and well-being apps for behavior change [85], similar BCTs for practice or rehearsal, self-monitoring, and prompts or reminders were reported as the most common. Health app users reported positively on the content and use of these apps [81]. However, there is limited evidence that they are the most effective in changing health or behavior [86].

Of the 125 combined BCTs and PSD elements that could have potentially been coded in the 23 interventions represented in this scoping review, 40% (50/125) were not represented in the text (n=48, 38.4% BCTs and n=2, 1.6% PSD elements). The following BCTs that may be effective in sustaining new behaviors or developing new habits were not included in any of the parent-focused eHealth interventions included in this review: *Habit Formation*, *Valued Self-Identity*, *Identity* *Associated with Changed Behaviour*, and *Focus on Past Success*. Similarly, the PSD elements *Social Comparison* and *Recognition* were not coded in any intervention despite their potential for effectively persuading behavior change through social influence. Exploring BCTs and PSD elements that are less familiar may be helpful for increasing parent-focused eHealth effectiveness. These less commonly used BCTs and PSD elements have been used successfully in other apps. For example, Runkeeper is a health and fitness app where runners can post their workouts and compare them with those of selected friends (social comparison), and Fitocracy is a digital health and fitness app that publishes the names of people who have achieved fitness milestones on the website’s fan page (recognition) [87]. Even BCTs that seem negative and incompatible with parent-focused eHealth, such as the BCTs *Future Punishment* and *Imaginary Punishment*, have been used effectively in other health apps. In a study looking at decreasing sedentary time by standing up, researchers found negative reinforcement to be more persuasive than constantly positive messaging. Negative reinforcement was described as showing a warning on the app if the user did not respond to the standing prompt. “Punishment” was applied using an avatar that became distressed and progressively more damaged in response to the user not standing when prompted [88].

Designing interventions based predominantly on what is commonly found in existing interventions or what people say they want is a common and common-sense approach to designing eHealth interventions but may miss important subconscious and reflexive drivers of sustained health behavior change. Taxonomies provide a rich catalog of possible BCTs and persuasive elements that are yet to be explored fully in parent-focused eHealth. Designing eHealth interventions to intentionally test BCTs and PSD elements that are less common and familiar could lead to important insights into the usability and effectiveness of digital interventions to support parents of children with chronic health care needs.

### Limitations

This scoping review only included English-language publications from before 2018. The COVID-19 pandemic has resulted in rapid shifts in web-based therapy provision that may not be described adequately in this review. However, capturing a decade of work before the COVID-19 pandemic also provides a useful baseline for future comparisons of change. In addition, the objective of this scoping review was to explore the utility of 2 taxonomies in defining the active ingredients and features of an intervention. The BCTTv1 and PSDM have not been updated since 2017. The data from the included articles provided sufficient information to ascertain the value of using both taxonomies in their entirety rather than merging or condensing them, as has occurred in other publications. Retrospective coding of publications based on taxonomy definitions was noted to be prone to errors, as has been reported elsewhere [14,89,90]. Similar errors may have occurred during our coding process. Explanations as to how we interpreted definitions have been included in the *Methods* section to increase transparency and decrease ambiguity where possible.

### Comparison With Related Reviews

A total of 3 other scoping reviews related to the scope of this review have been published previously [91-93]. These publications did not address the objectives of this scoping review to map the active ingredients and intervention features in parent-focused eHealth and explore the use of both taxonomies. Greffin and Barros [91] investigated health-related parenting but did not address active ingredients and intervention features of behavior change and persuasive technology. Bradshaw et al [92] reviewed behavior change in parent-focused interventions but did not limit their review to eHealth and did not review persuasive technology. Asbjørnsen et al [93] looked at both behavior change and persuasive technology; however, the focus was on weight loss maintenance in adults. In addition, the authors used the 16 BCTTv1 categories rather than coding all 93 BCTs.

### Conclusions

The 2 taxonomies representing behavior change and persuasive technology were found to code different constructs, discouraging merging or reduction. Merging taxonomies may fail to capture the persuasive ingredients inherent in technology, missing the unique role of technology in creating bonding relationships with the end user. Using both taxonomies to code interventions in this scoping review was useful in providing a comprehensive description of the active ingredients and features of an eHealth intervention. However, it is important to note that potential coding errors may render the accuracy of the description uncertain. Coding errors may result from limited space for comprehensive reporting of the active ingredients and intervention features or from ambiguity in the publications’ description of ingredients, features, behavior targets, and outcomes.

Commonly found BCTs and PSD elements did not necessarily represent the best BCTs or PSD elements to use, with some potentially impactful codes not being used at all in the parent-focused eHealth data set. Despite the time-consuming and cumbersome task of capturing all BCTs and PSD elements, identifying each of these constructs enables a much richer description of the ingredients within the interventions as well as providing opportunities to consider innovative approaches in future designs. This scoping review highlighted the need for supplementary material detailing the active ingredients and intervention features in publications and the benefit of using 2 taxonomies to address both behavior change and persuasive technology that together influence the experience and behaviors of the people using the technology.

## Appendix

Multimedia Appendix 1 Search terms for Scopus.

Multimedia Appendix 2 Persuasive system design (PSD) element frequency of use: number of interventions coded per PSD element.

Multimedia Appendix 3 Behavior change technique (BCT) frequency of use: number of interventions coded per BCT.

## Abbreviations

BCT: behavior change technique
BCTTv1: Behavior Change Technique Taxonomy version 1
COM-B: Capability, Opportunity, and Motivation–Behavior
FBM: Fogg Behavior Model
PRISMA-ScR: Preferred Reporting Items for Systematic Reviews and Meta-Analyses extension for Scoping Reviews
PSD: persuasive system design
PSDM: Persuasive System Design Model

## References

[1] Conner M, Norman P (2017). Health behaviour: current issues and challenges. Psychol Health.

[2] Michie S, Johnston M, Francis J, Hardeman W, Eccles M (2008). From theory to intervention: mapping theoretically derived behavioural determinants to behaviour change techniques. Appl Psychol.

[3] Oinas-Kukkonen H, Harjumaa M (2008). A systematic framework for designing and evaluating persuasive systems. Proceedings of the PERSUASIVE 2008: Persuasive Technology.

[4] Ashton LM, Sharkey T, Whatnall MC, Williams RL, Bezzina A, Aguiar EJ, Collins CE, Hutchesson MJ (2019). Effectiveness of interventions and behaviour change techniques for improving dietary intake in young adults: a systematic review and meta-analysis of RCTs. Nutrients.

[5] Kelders SM, Kok RN, Ossebaard HC, Van Gemert-Pijnen JE (2012). Persuasive system design does matter: a systematic review of adherence to web-based interventions. J Med Internet Res.

[6] Lentferink AJ, Oldenhuis HK, de Groot M, Polstra L, Velthuijsen H, van Gemert-Pijnen JE (2017). Key components in eHealth interventions combining self-tracking and persuasive eCoaching to promote a healthier lifestyle: a scoping review. J Med Internet Res.

[7] Samdal GB, Eide GE, Barth T, Williams G, Meland E (2017). Effective behaviour change techniques for physical activity and healthy eating in overweight and obese adults; systematic review and meta-regression analyses. Int J Behav Nutr Phys Act.

[8] Willett M, Duda J, Fenton S, Gautrey C, Greig C, Rushton A (2019). Effectiveness of behaviour change techniques in physiotherapy interventions to promote physical activity adherence in lower limb osteoarthritis patients: a systematic review. PLoS One.

[9] Lehto T, Oinas-Kukkonen H (2011). Persuasive features in web-based alcohol and smoking interventions: a systematic review of the literature. J Med Internet Res.

[10] Geuens J, Swinnen TW, Westhovens R, de Vlam K, Geurts L, Vanden Abeele V (2016). A review of persuasive principles in mobile apps for chronic arthritis patients: opportunities for improvement. JMIR Mhealth Uhealth.

[11] Klaassen R, Bul KC, Op den Akker R, van der Burg GJ, Kato PM, Di Bitonto P (2018). Design and evaluation of a pervasive coaching and gamification platform for young diabetes patients. Sensors (Basel).

[12] Wang Y, Fadhil A, Lange J, Reiterer H (2019). Integrating taxonomies into theory-based digital health interventions for behavior change: a holistic framework. JMIR Res Protoc.

[13] Wood CE, Hardeman W, Johnston M, Francis J, Abraham C, Michie S (2016). Reporting behaviour change interventions: do the behaviour change technique taxonomy v1, and training in its use, improve the quality of intervention descriptions?. Implement Sci.

[14] Johnston M, Johnston D, Wood CE, Hardeman W, Francis J, Michie S (2018). Communication of behaviour change interventions: can they be recognised from written descriptions?. Psychol Health.

[15] Ogden J (2016). Celebrating variability and a call to limit systematisation: the example of the Behaviour Change Technique Taxonomy and the Behaviour Change Wheel. Health Psychol Rev.

[16] Conner M, Norman P (2015). Predicting and Changing Health Behaviour: Research and Practice with Social Cognition Models.

[17] Interventions. BCT Taxonomy v1.

[18] Fogg BJ, Drew J (2003). Persuasive Technology.

[19] Chen YX, Liang HW, Lin PH, Hsia J-C, Chang C-H, Wang C-Y, Hung Y-P (2017). Dynamic text messaging in a persuasive gaming environment to promote participation in self-tracking of health status. Proceedings of the Fifth International Workshop on Behavior Change Support Systems (BCSS’17).

[20] Head KJ, Noar SM, Iannarino NT, Grant Harrington N (2013). Efficacy of text messaging-based interventions for health promotion: a meta-analysis. Soc Sci Med.

[21] Ritterband LM, Thorndike FP, Cox DJ, Kovatchev BP, Gonder-Frederick LA (2009). A behavior change model for internet interventions. Ann Behav Med.

[22] Colquhoun HL, Levac D, O'Brien KK, Straus S, Tricco AC, Perrier L, Kastner M, Moher D (2014). Scoping reviews: time for clarity in definition, methods, and reporting. J Clin Epidemiol.

[23] Colquhoun HL, Jesus TS, O'Brien KK, Tricco AC, Chui A, Zarin W, Lillie E, Hitzig SL, Straus S (2017). Study protocol for a scoping review on rehabilitation scoping reviews. Clin Rehabil.

[24] Arksey H, O'Malley L (2005). Scoping studies: towards a methodological framework. Int J Social Res Methodol.

[25] Munn Z, Peters MD, Stern C, Tufanaru C, McArthur A, Aromataris E (2018). Systematic review or scoping review? Guidance for authors when choosing between a systematic or scoping review approach. BMC Med Res Methodol.

[26] Tricco AC, Lillie E, Zarin W, O'Brien KK, Colquhoun H, Levac D, Moher D, Peters MD, Horsley T, Weeks L, Hempel S, Akl EA, Chang C, McGowan J, Stewart L, Hartling L, Aldcroft A, Wilson MG, Garritty C, Lewin S, Godfrey CM, Macdonald MT, Langlois EV, Soares-Weiser K, Moriarty J, Clifford T, Tunçalp Ö, Straus SE (2018). PRISMA extension for scoping reviews (PRISMA-ScR): checklist and explanation. Ann Intern Med.

[27] Saquetto MB, de Santana Bispo A, da Silva Barreto C, Gonçalves KA, Queiroz RS, da Silva CM, Gomes Neto M (2018). Addition of an educational programme for primary caregivers to rehabilitation improves self-care and mobility in children with cerebral palsy: a randomized controlled trial. Clin Rehabil.

[28] McPherson M, Arango P, Fox H, Lauver C, McManus M, Newacheck PW, Perrin JM, Shonkoff JP, Strickland B (1998). A new definition of children with special health care needs. Pediatrics.

[29] Sims R (1997). Interactivity: a forgotten art?. Comput Human Behav.

[30] Hall CM, Bierman KL (2015). Technology-assisted interventions for parents of young children: emerging practices, current research, and future directions. Early Child Res Q.

[31] Jones DJ (2014). Future directions in the design, development, and investigation of technology as a service delivery vehicle. J Clin Child Adolesc Psychol.

[32] Michie S, Yardley L, West R, Patrick K, Greaves F (2017). Developing and evaluating digital interventions to promote behavior change in health and health care: recommendations resulting from an international workshop. J Med Internet Res.

[33] Levac D, Colquhoun H, O'Brien KK (2010). Scoping studies: advancing the methodology. Implement Sci.

[34] Silva ME, Graham F, Levack W, Hay-Smith J (2019). Persuasive technology and behaviour change in parent-focused eHealth interventions supporting child health: a scoping review protocol. New Zealand J Physiother.

[35] The Joanna Briggs Institute (2015). Methodology for JBI scoping reviews. Joanna Briggs Institute Reviewers' Manual: 2015 edition / Supplement.

[36] Gustafson SL, Rhodes RE (2006). Parental correlates of physical activity in children and early adolescents. Sports Med.

[37] Pietilä A, Nurmi S, Halkoaho A, Kyngäs H (2020). Qualitative research: ethical considerations. The Application of Content Analysis in Nursing Science Research.

[38] Hay-Smith EJ, Englas K, Dumoulin C, Ferreira CH, Frawley H, Weatherall M (2019). The Consensus on Exercise Reporting Template (CERT) in a systematic review of exercise-based rehabilitation effectiveness: completeness of reporting, rater agreement, and utility. Eur J Phys Rehabil Med.

[39] Hay-Smith J, Peebles L, Farmery D, Dean S, Grainger R (2019). Apps-olutely fabulous? - The quality of PFMT smartphone app content and design rated using the Mobile App Rating Scale, Behaviour Change Taxonomy, and guidance for exercise prescription. Proceedings of the ICS 2019 Gothenburg Scientific Programme.

[40] Hay-Smith E, McClurg D, Frawley H, Dean S (2016). Exercise adherence: integrating theory, evidence and behaviour change techniques. Physiotherapy.

[41] (2014). Starter Pack For trainees. BCT Taxonomy.

[42] Fogg BJ (2018). Behaviour Design Guidebook.

[43] Wang J, Yao N, Shen M, Zhang X, Wang Y, Liu Y, Geng Z, Yuan C (2016). Supporting caregivers of children with acute lymphoblastic leukemia via a smartphone app: a pilot study of usability and effectiveness. Comput Inform Nurs.

[44] Wang J, Yao N, Wang Y, Zhou F, Liu Y, Geng Z, Yuan C (2015). Developing “care assistant”: a smartphone application to support caregivers of children with acute lymphoblastic leukaemia. J Telemed Telecare.

[45] Waligórska A, Pisula E, Waligórski M, Letachowicz M (2012). AutismPro system in supporting treatment of children with autism in Poland. Pediatr Int.

[46] Wiecha JM, Adams WG, Rybin D, Rizzodepaoli M, Keller J, Clay JM (2015). Evaluation of a web-based asthma self-management system: a randomised controlled pilot trial. BMC Pulm Med.

[47] Donovan CL, March S (2014). Online CBT for preschool anxiety disorders: a randomised control trial. Behav Res Ther.

[48] March S, Spence SH, Donovan CL (2009). The efficacy of an internet-based cognitive-behavioral therapy intervention for child anxiety disorders. J Pediatr Psychol.

[49] Spence SH, Donovan CL, March S, Kenardy JA, Hearn CS (2017). Generic versus disorder specific cognitive behavior therapy for social anxiety disorder in youth: a randomized controlled trial using internet delivery. Behav Res Ther.

[50] Gustafson D, Wise M, Bhattacharya A, Pulvermacher A, Shanovich K, Phillips B, Lehman E, Chinchilli V, Hawkins R, Kim J (2012). The effects of combining Web-based eHealth with telephone nurse case management for pediatric asthma control: a randomized controlled trial. J Med Internet Res.

[51] Morgan AJ, Rapee RM, Tamir E, Goharpey N, Salim A, McLellan LF, Bayer JK (2015). Preventing anxiety problems in children with Cool Little Kids Online: study protocol for a randomised controlled trial. Trials.

[52] Morgan AJ, Rapee RM, Salim A, Goharpey N, Tamir E, McLellan LF, Bayer JK (2017). Internet-delivered parenting program for prevention and early intervention of anxiety problems in young children: randomized controlled trial. J Am Acad Child Adolesc Psychiatry.

[53] Morgan AJ, Rapee RM, Salim A, Bayer JK (2018). Predicting response to an internet-delivered parenting program for anxiety in early childhood. Behav Ther.

[54] Nkoy FL, Stone BL, Fassl BA, Koopmeiners K, Halbern S, Kim EH, Poll J, Hales JW, Lee D, Maloney CG (2012). Development of a novel tool for engaging children and parents in asthma self-management. AMIA Annu Symp Proc.

[55] Breitenstein SM, Shane J, Julion W, Gross D (2015). Developing the eCPP: adapting an evidence-based parent training program for digital delivery in primary care settings. Worldviews Evid Based Nurs.

[56] Ibañez LV, Kobak K, Swanson A, Wallace L, Warren Z, Stone WL (2018). Enhancing interactions during daily routines: a randomized controlled trial of a web-based tutorial for parents of young children with ASD. Autism Res.

[57] Turner K, Reynolds JN, McGrath P, Lingley-Pottie P, Huguet A, Hewitt A, Green C, Wozney L, Mushquash C, Muhajarine N, Sourander A, Caughey H, Roane J (2015). Guided internet-based parent training for challenging behavior in children with fetal alcohol spectrum disorder (strongest families FASD): study protocol for a randomized controlled trial. JMIR Res Protoc.

[58] Jones DJ, Forehand R, Cuellar J, Parent J, Honeycutt A, Khavjou O, Gonzalez M, Anton M, Newey GA (2014). Technology-enhanced program for child disruptive behavior disorders: development and pilot randomized control trial. J Clin Child Adolesc Psychol.

[59] Zhang FF, Meagher S, Scheurer M, Folta S, Finnan E, Criss K, Economos C, Dreyer Z, Kelly M (2016). Developing a web-based weight management program for childhood cancer survivors: rationale and methods. JMIR Res Protoc.

[60] Curtis KE, Lahiri S, Brown KE (2015). Targeting parents for childhood weight management: development of a theory-driven and user-centered healthy eating app. JMIR Mhealth Uhealth.

[61] Gerencser KR, Higbee TS, Akers JS, Contreras BP (2017). Evaluation of interactive computerized training to teach parents to implement photographic activity schedules with children with autism spectrum disorder. J Appl Behav Anal.

[62] Ingersoll B, Berger NI (2015). Parent engagement with a telehealth-based parent-mediated intervention program for children with autism spectrum disorders: predictors of program use and parent outcomes. J Med Internet Res.

[63] Ingersoll B, Shannon K, Berger N, Pickard K, Holtz B (2017). Self-directed telehealth parent-mediated intervention for children with autism spectrum disorder: examination of the potential reach and utilization in community settings. J Med Internet Res.

[64] Van Eerdenbrugh S, Packman A, Onslow M, O'brian S, Menzies R (2017). Development of an internet version of the Lidcombe Program of early stuttering intervention: a trial of Part 1. Int J Speech Lang Pathol.

[65] Christakis DA, Garrison MM, Lozano P, Meischke H, Zhou C, Zimmerman FJ (2012). Improving parental adherence with asthma treatment guidelines: a randomized controlled trial of an interactive website. Acad Pediatr.

[66] Meischke H, Lozano P, Zhou C, Garrison MM, Christakis D (2011). Engagement in "My Child's Asthma", an interactive web-based pediatric asthma management intervention. Int J Med Inform.

[67] Högström Jens, Enebrink P, Ghaderi A (2013). The moderating role of child callous-unemotional traits in an internet-based parent-management training program. J Fam Psychol.

[68] Gal E, Steinberg O (2018). Using home-program adherence app in pediatric therapy: case study of sensory processing disorder. Telemed J E Health.

[69] Hammersley ML, Jones RA, Okely AD (2017). Time2bHealthy - An online childhood obesity prevention program for preschool-aged children: a randomised controlled trial protocol. Contemp Clin Trials.

[70] Baker S, Sanders MR, Turner KM, Morawska A (2017). A randomized controlled trial evaluating a low-intensity interactive online parenting intervention, Triple P Online Brief, with parents of children with early onset conduct problems. Behav Res Ther.

[71] Dittman CK, Farruggia SP, Palmer ML, Sanders MR, Keown LJ (2014). Predicting success in an online parenting intervention: the role of child, parent, and family factors. J Fam Psychol.

[72] Sanders MR, Dittman CK, Farruggia SP, Keown LJ (2014). A comparison of online versus workbook delivery of a self-help positive parenting program. J Prim Prev.

[73] Gabrielli S, Dianti M, Maimone R, Betta M, Filippi L, Ghezzi M, Forti S (2017). Design of a mobile app for nutrition education (TreC-LifeStyle) and formative evaluation with families of overweight children. JMIR Mhealth Uhealth.

[74] Chifari A, Sílvia A, Panagiotis B (2013). WHAAM: Web Health Application for Adhd Monitoring - Context-Driven Framework. The WHAAM project.

[75] Merlo G, Chiazzese G, Sanches-Ferreira M, Chifari A, Seta L, McGee C, Mirisola A, Giammusso I (2018). The WHAAM application: a tool to support the evidence-based practice in the functional behaviour assessment. J Innov Health Inform.

[76] Silvia A, Bamidis PD, Bilbow A, Chiazzese G, Antonella C, Paola D, Di Giuseppe O, Gavin D, Mcgee C, Merlo G, Sanches-Ferreira M, Santos MA, Monica S-M, Dimitris S (2015). WHAAM: A New Online Service Supporting Parents and Teachers of Children With ADHD.

[77] Spachos D, Chifari A, Chiazzese G, Merlo G, Doherty G, Bamidis P (2014). WHAAM: a mobile application for ubiquitous monitoring of ADHD behaviors. Proceedings of the International Conference on Interactive Mobile Communication Technologies and Learning (IMCL2014).

[78] Michie S, Atkins L, West R (2014). The Behaviour Change Wheel A Guide to Designing Interventions.

[79] Brigden A, Anderson E, Linney C, Morris R, Parslow R, Serafimova T, Smith L, Briggs E, Loades M, Crawley E (2020). Digital behavior change interventions for younger children with chronic health conditions: systematic review. J Med Internet Res.

[80] Orji R, Moffatt K (2018). Persuasive technology for health and wellness: state-of-the-art and emerging trends. Health Informatics J.

[81] Wiafe I, Nakata K (2012). Trends in persuasive systems design: techniques, methods and application domain. Int J Societal Appl Comput Sci.

[82] Tate DF, Lytle LA, Sherwood NE, Haire-Joshu D, Matheson D, Moore SM, Loria CM, Pratt C, Ward DS, Belle SH, Michie S (2016). Deconstructing interventions: approaches to studying behavior change techniques across obesity interventions. Transl Behav Med.

[83] Wildeboer G, Kelders SM, van Gemert-Pijnen JE (2016). The relationship between persuasive technology principles, adherence and effect of web-based interventions for mental health: a meta-analysis. Int J Med Inform.

[84] Wiart L, Ray L, Darrah J, Magill-Evans J (2010). Parents' perspectives on occupational therapy and physical therapy goals for children with cerebral palsy. Disabil Rehabil.

[85] McKay FH, Wright A, Shill J, Stephens H, Uccellini M (2019). Using health and well-being apps for behavior change: a systematic search and rating of apps. JMIR Mhealth Uhealth.

[86] Milne-Ives M, Lam C, De Cock C, Van Velthoven MH, Meinert E (2020). Mobile apps for health behavior change in physical activity, diet, drug and alcohol use, and mental health: systematic review. JMIR Mhealth Uhealth.

[87] Yoganathan D, Kajanan S (2013). Persuasive technology for smartphone fitness apps. Proceedings of the Pacific Asia Conference on Information Systems 2013.

[88] Aydin A, Girouard A (2018). Couch: investigating the relationship between aesthetics and persuasion in a mobile application. Proceedings of the 44th Graphics Interface Conference.

[89] Byrne M (2020). Gaps and priorities in advancing methods for health behaviour change research. Health Psychol Rev.

[90] Knittle K (2015). We cannot keep firing blanks - yet another appeal for improved RCT reporting: commentary on Peters, de Bruin and Crutzen. Health Psychol Rev.

[91] Barros L, Greffin K (2017). Supporting health-related parenting: a scoping review of programs assisted by the internet and related technologies. Estud psicol (Campinas).

[92] Bradshaw SR, Shaw K, Bem D, Cummins C (2017). Improving health, well-being and parenting skills in parents of children with medical complexity: a scoping review protocol. BMJ Open.

[93] Asbjørnsen RA, Smedsrød ML, Solberg Nes L, Wentzel J, Varsi C, Hjelmesæth J, van Gemert-Pijnen JE (2019). Persuasive system design principles and behavior change techniques to stimulate motivation and adherence in electronic health interventions to support weight loss maintenance: scoping review. J Med Internet Res.

