# Supplementary material for: Exploring the Use of the Behavior Change Technique Taxonomy and the Persuasive System Design Model in Defining Parent-Focused eHealth Interventions: Scoping Review
Source: J Med Internet Res. 2023 Jun 21;25:e42083. doi: 10.2196/42083 (PMC10337339; doi:10.2196/42083)
Supplement: Multimedia Appendix 1 [file jmir_v25i1e42083_app1.doc]

| Search Parameters | Keywords |
| --- | --- |
|  |  |
| Technology | Mobile Applications; Internet; social media; telerehabilitation; Telemedicine; computers, handheld/ or smartphone/  ("Persuasive System*" or "behav* change support system*" or captology or "human computer interface" or human-computer interface)  ("information technolog*" or "smart phone*" or app or apps or computer* or e-health or ehealth or internet* or ipad* or iphone* or i-phone* or i-pad* or m-health or mhealth or mobile or online* or persuasive or smart-phone or smartphone* or "tablet computer" or technolog* or telecare or telehealth or telemedic* or telemonitoring or telerehabilitation or "web based" or "web-based" or website*) |
| Parents | Caregivers; family; parent/ or father/ or mother/ or single parent/ parent-child relations/ or father-child relations/ or mother-child relations/ or parenting/; Child Rearing/  (caregiver* or parent* or mother* or mom* or mum* or father* or dad* or famil*)  ("parent focused" or parent-focused or "parent* of children" or "parent* of a child |
| Paediatric population / cerebral palsy | Child Development; Pediatrics; child behavior ; child health/ Child Care; child health services/ or "early intervention (education)"/; cerebral palsy/ child/ or disabled children/  (child* or pediatric* or paediatric* or "cerebral pals*" or "child* adj4 disab*") |
| Home exercise programmes and rehabilitation | patient compliance; practice guideline; Health Behavior; tertiary prevention; Muscle Stretching Exercises/ or Exercise Therapy/; Exercise; Health Education/ or Consumer Health Information/ or Patient Education as Topic; or physical fitness/ or health communication/ or health promotion/ or healthy people programs/ or weight reduction programs; rehabilitation/ or "activities of daily living"/ or exercise therapy/ or neurological rehabilitation/ or occupational therapy/ or "rehabilitation of speech and language disorders"/; Patient Care/; Posture/ or Patient Positioning  (resource* or "problem solv" or "problem-solv*" or "goal set*" or "goal-set" or help* or improv* or reduc* or develop* or increas* or impact* or adher* or compliance or comply or complies or care* or caring or portal* or platform* or home* or persuasive or train* or educat* or change or promot* or rehab* or treat* or serv* or support* or motivat* or coach or inform* or health or manag* or behav* or interven* or prevent* or program* or physical or sedentary or excercis* or therap* or physiotherap* or lifestyle or life-style* or tutor*) |
| Words in brackets used as .mp search | [mp=title, abstract, original title, name of substance word, subject heading word, keyword heading word, protocol supplementary concept word, rare disease supplementary concept word, unique identifier, synonyms] |
