# Supplementary figures and images for: Exploring the Use of the Behavior Change Technique Taxonomy and the Persuasive System Design Model in Defining Parent-Focused eHealth Interventions: Scoping Review
Source: J Med Internet Res. 2023 Jun 21;25:e42083. doi: 10.2196/42083 (PMC10337339; doi:10.2196/42083)

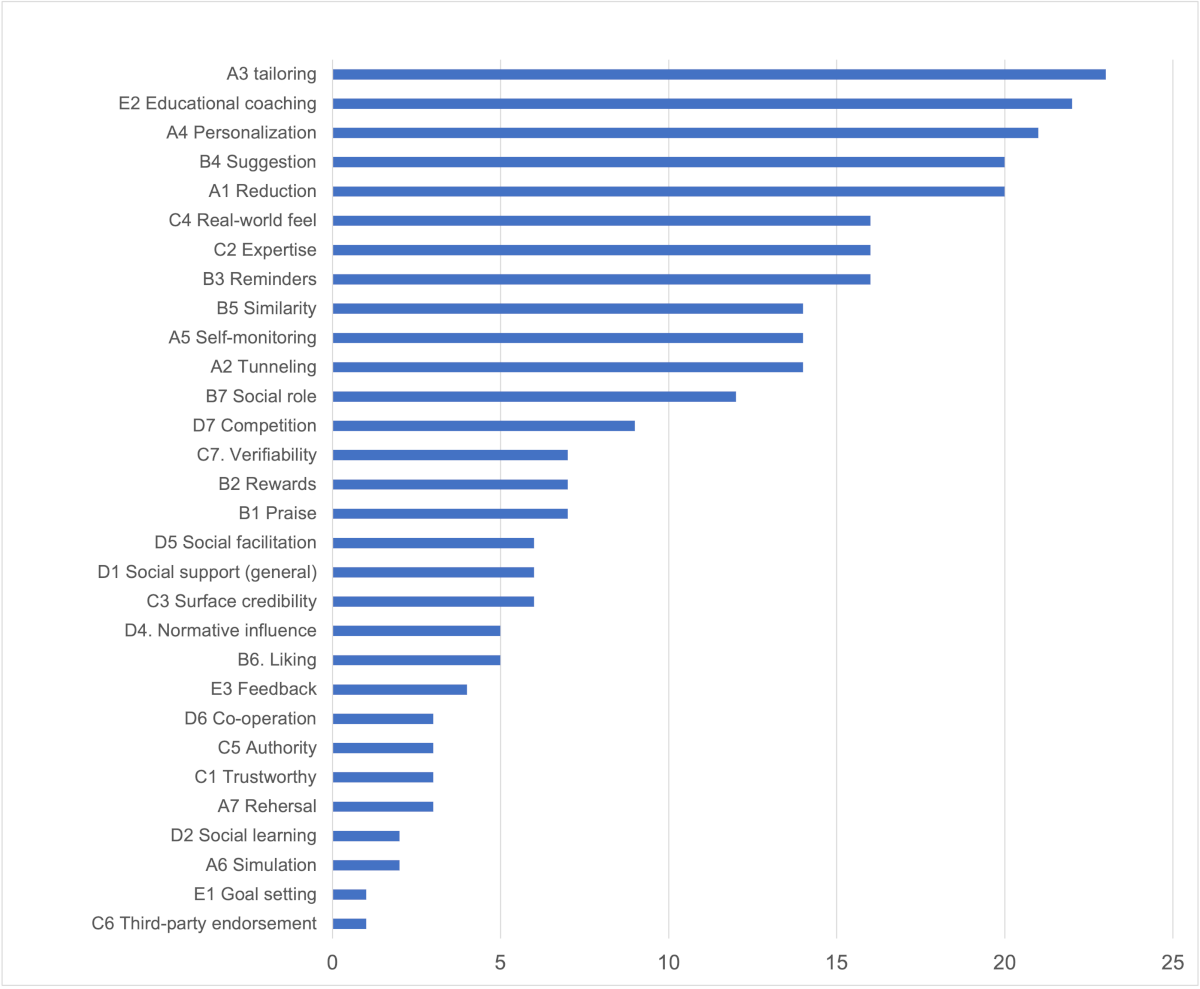

Supplement: Multimedia Appendix 2 [file jmir_v25i1e42083_app2.png]

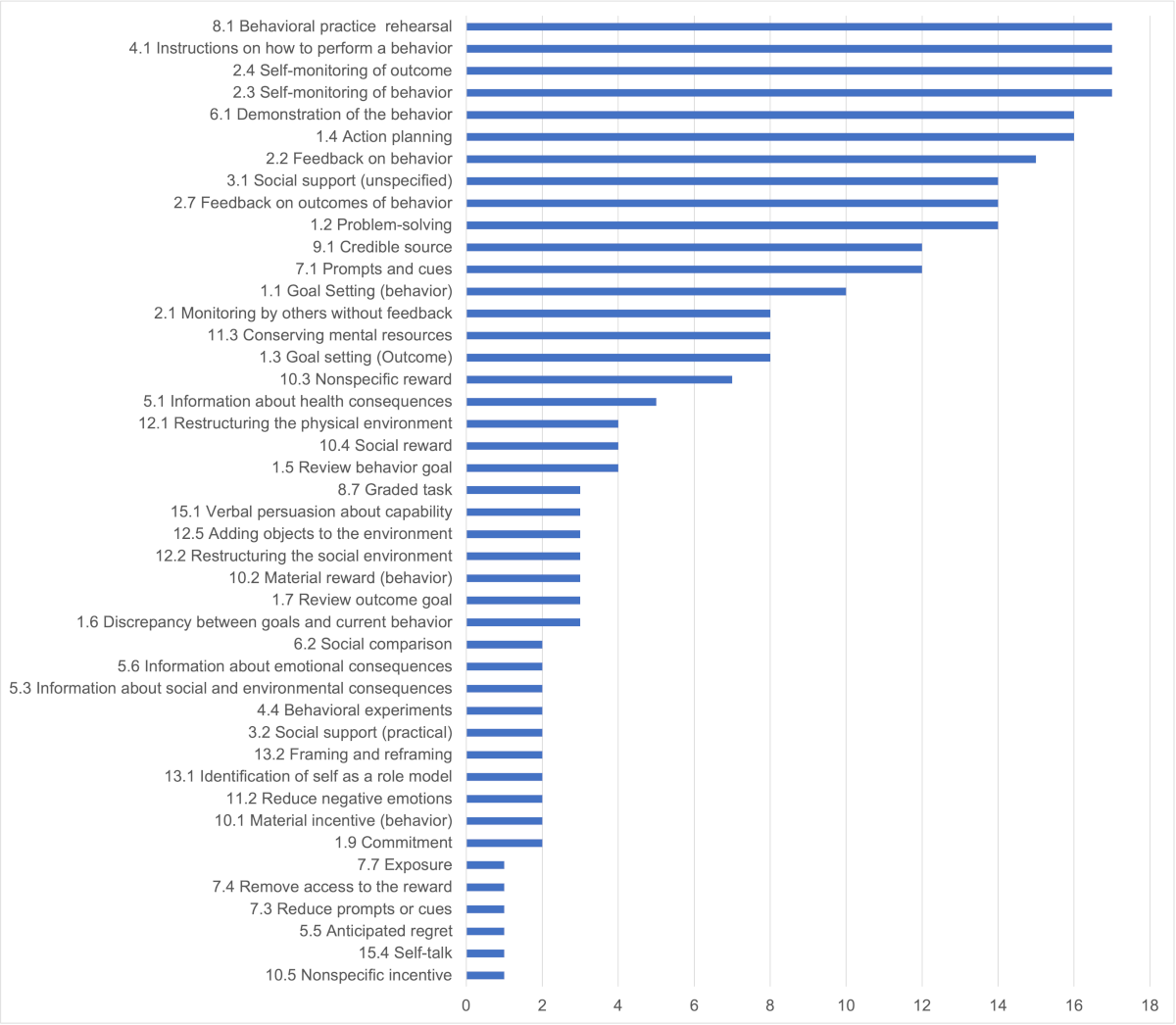

Supplement: Multimedia Appendix 3 [file jmir_v25i1e42083_app3.png]
